# Activation of Melanocortin-4 Receptor Inhibits Both Neuroinflammation Induced by Early Exposure to Ethanol and Subsequent Voluntary Alcohol Intake in Adulthood in Animal Models: Is BDNF the Key Mediator?

**DOI:** 10.3389/fncel.2020.00005

**Published:** 2020-01-28

**Authors:** Osvaldo Flores-Bastías, Alfredo Adriasola-Carrasco, Eduardo Karahanian

**Affiliations:** ^1^Institute of Biomedical Sciences, Faculty of Health Sciences, Universidad Autónoma de Chile, Santiago, Chile; ^2^Research Center for the Study of Alcohol Drinking Behavior in Adolescents, Universidad Autónoma de Chile, Santiago, Chile

**Keywords:** melanocyte-stimulating hormone, MC4R, brain-derived neurotrophic factor, alcohol use disorder, alcoholism, α-MSH

## Abstract

The concept that neuroinflammation induced by excessive alcohol intake in adolescence triggers brain mechanisms that perpetuate consumption has strengthened in recent years. The melanocortin system, composed of the melanocortin 4 receptor (MC4R) and its ligand α-melanocyte-stimulating hormone (α-MSH), has been implicated both in modulation of alcohol consumption and in ethanol-induced neuroinflammation decrease. Chronic alcohol consumption in adolescent rats causes a decrease in an α-MSH release by the hypothalamus, while the administration of synthetic agonists of MC4R causes a decrease in neuroinflammation and a decrease in voluntary alcohol consumption. However, the mechanism that connects the activation of MC4R with the decrease of both neuroinflammation and voluntary alcohol consumption has not been elucidated. Brain-derived neurotrophic factor (BDNF) has been implicated in alcohol drinking motivation, dependence and withdrawal, and its levels are reduced in alcoholics. Deficiencies in BDNF levels increased ethanol self-administration in rats. Further, BDNF triggers important anti-inflammatory effects in the brain, and this could be one of the mechanisms by which BDNF reduces chronic alcohol intake. Interestingly, MC4R signaling induces BDNF expression through the activation of the cAMP-responsive element-binding protein (CREB). We hypothesize that ethanol exposure during adolescence decreases the expression of α-MSH and hence MC4R signaling in the hippocampus, leading to a lower BDNF activity that causes dramatic changes in the brain (e.g., neuroinflammation and decreased neurogenesis) that predispose to maintain alcohol abuse until adulthood. The activation of MC4R either by α-MSH or by synthetic agonist peptides can induce the expression of BDNF, which would trigger several processes that lead to lower alcohol consumption.

Alcohol is the most commonly abused drug worldwide. The World Health Organization (WHO) placed alcohol abuse disorder (AUD) among the top risk factors for diseases leading to death worldwide (Lim et al., 2012; World Health Organization, 2014). As reported by WHO, the global effect of alcohol abuse is near 3.3 million deaths per year (approximately 5.9% of all deaths worldwide; World Health Organization, 2014). A study in the United Kingdom concluded that alcohol is the most noxious drug to society, above heroin and cocaine (Nutt et al., 2010). Alcohol abuse is associated with an increased risk of developing a variety of health issues including cancer and liver cirrhosis (Baan et al., 2007; Shield et al., 2013).

In addition to the various health problems associated with AUD, the excessive consumption of alcohol generates significant consequences in the brain such as neurodegeneration (Crews and Nixon, 2009), depression (Whiteford et al., 2013), anxiety (Kushner et al., 2000), memory impairment (Rose and Grant, 2010) and malfunction of the prefrontal cortex that leads to impaired executive function (e.g., planning, thinking, and judgment; Peterson et al., 1990). These alterations are especially important if excessive alcohol consumption occurs during brain development in adolescence, which would lead to long-term damage to brain structure and function (Crews et al., 2007). There is extensive evidence supporting the idea that the beginning of alcohol consumption in adolescence increases the risk of developing AUD in adulthood (DeWit et al., 2000; Dawson et al., 2008).

Although there are many neurobiological mechanisms involved in the development of AUD (i.e., motivation for alcohol consumption, establishment of chronic alcohol intake and relapse), the idea that neuroinflammation and oxidative stress play a role in the maintenance of chronic alcohol intake has recently gained relevance (Israel et al., 2017). It has been proposed that the activation of genes related to the innate immune system in the brain and neuroinflammation play a significant role in the establishment of addiction to alcohol (Coller and Hutchinson, 2012; Flores-Bastías and Karahanian, 2018). The activation of neuroinflammation would produce long-lasting neurobiological changes, as increased glutamate-induced hyperexcitability and excitotoxicity and decreased neurogenesis, which are related to addictive substances abuse; all these neurobiological changes are more evident if alcohol abuse begins at an early age (Crews et al., 2011). Although it was already known that alcohol consumption produced neuroinflammation (Blanco and Guerri, 2007), the first evidence that neuroinflammation would be directly related to alcohol addiction came in 2011 from experiments by Blednov et al. (2011), who demonstrated that the systemic administration of bacterial lipopolysaccharide (LPS) to mice increases voluntary ethanol intake for a prolonged period. Ethanol consumption disturb the intestinal barrier, allowing the passage of bacterial LPS into circulation (Ferrier et al., 2006). LPS inside the body induces the synthesis of tumor necrosis factor-α (TNF-α) by Kupffer cells, which is released from liver to the blood; TNF-α then crosses the blood-brain barrier and activates microglia and astrocytes TNF receptors to induce neuroinflammation (Qin et al., 2007; Crews and Vetreno, 2016). This state of neuroinflammation is maintained for several months (Qin et al., 2007), insinuating that there exist long-lasting mechanisms that perpetuate it. Accordingly, ethanol-induced neuroinflammation persists upregulated for a long period after alcohol withdrawal, whereas levels of peripheral inflammatory cytokines rapidly return to basal, indicating that neuroinflammation is maintained by a strong mechanism of self-perpetuation (Qin et al., 2008).

Besides the neuroinflammatory processes triggered by systemic LPS, the generation of reactive oxygen species (ROS) from ethanol metabolism in the brain may also contribute to neuroinflammation (Flores-Bastías and Karahanian, 2018). Microglia and astrocytes are key cells in the neuroinflammatory process, and they are highly reactive to ethanol exposure (Orellana et al., 2017), releasing proinflammatory cytokines and nitric oxide (Blanco and Guerri, 2007). Ethanol intake produces a potent activation of glial cells in both adult and adolescent rodents. Interestingly, withdrawal of alcohol intake by adolescent rodents does not completely reverse such activation when they reach adulthood (Evrard et al., 2006). The hippocampus is the brain region that suffers major pathological alterations due to chronic alcohol consumption (Franke et al., 1997). Astrocytes and microglia are highly activated in the hippocampus from alcoholics (He and Crews, 2008), increasing the release of pro-inflammatory cytokines which can lead to neuron death (Ward et al., 2009). In the case of adolescents, their hippocampus suffers greater damage than that of adults, since they are more sensitive to the EtOH-induced alterations of memory processes requiring hippocampal integrity (Markwiese et al., 1998; White and Swartzwelder, 2004).

Microglia, astrocytes, and neurons abundantly express NF-κB, an essential transcription factor that activates the genes of the innate immune response. NF-κB is also involved in ethanol-induced neuroinflammation (Zou and Crews, 2010). Ethanol increases cytochrome P4502E1 (Cyp2E1) activity (Lieber, 1999), and in turn, Cyp2E1 oxidation of ethanol increases ROS levels that activate NF-κB directly (Chandel et al., 2000), leading to the synthesis of pro-inflammatory cytokines such as IL-6, IL-1β, and TNF-α (Cao et al., 2005). In addition, NF-κB activation induces NADPH oxidase expression (Cao et al., 2005), leading to the production of more ROS, which creates an “activation loop” that potentiates the innate immune response (Crews et al., 2011; Flores-Bastías and Karahanian, 2018). Accordingly, the daily administration of N-acetyl cysteine (a precursor in the formation of the antioxidant glutathione) to rats that have chronically consumed ethanol, has been shown to significantly reduce voluntary ethanol intake (Israel et al., 2017). Along this line, ibudilast, a drug that reduces TNF-α activity, inhibits by 50% chronic ethanol intake in high alcohol drinking rats (Bell et al., 2015). In addition to the intrinsic damage induced by ROS, leading to neuron death, NF-κB activation increase TNF-α, IL-1β, IL-6 and transforming growth factor β1 (TGF-β1) release, resulting in neuronal apoptosis (Crews et al., 2015). “The constant activation of neuroinflammation, together with the exacerbated production of ROS, would lead to neurodegeneration of key areas involved in excessive alcohol consumption. It has been proposed that, instead of simply being a side effect of excessive alcohol consumption, neuronal damage associated with drinking may actually underlie some of the mechanisms that regulate the development of alcohol abuse disorder (Crews et al., 2015). Alcohol-induced cell death in regions such as the prefrontal cortex may lead to a lack of inhibition in subcortical reward areas such as the striatum, which in turn may reduce behavioral inhibition and increase motivation to drink. Repeated stimulation of the innate immune system during chronic or binge alcohol consumption may facilitate this process by decreasing inhibition of the mesolimbic reward system, thus increasing drinking (Crews et al., 2011, 2015; Flores-Bastías and Karahanian, 2018).

The hypothalamus is one of the brain regions that undergoes more pathophysiological changes during excessive alcohol intake (Barson and Leibowitz, 2016). In the hypothalamus, several orexigenic peptides (such as orexin, encephalin, and galanin) stimulate alcohol consumption (Rada et al., 2004; Schneider et al., 2007; Barson et al., 2010); on the other hand, anorexigenic hormones such as dynorphin, corticotropin-releasing factor and melanocortins inhibit alcohol drinking (Thorsell et al., 2005; Navarro et al., 2008; Barson et al., 2010). Melanocortins (MC) is a group of neuropeptides that are produced in the arcuate nucleus of the hypothalamus (Arc) and that include α-, β-, and γ- melanocyte-stimulating hormones (α-MSH, β-MSH and γ-MSH; Hadley and Haskell-Luevano, 1999). α-MSH is involved in the regulation of sexual behavior, food appetite and memory through its agonist activity on MC3R and melanocortin 4 receptor (MC4R) melanocortin receptors present in the hippocampus, paraventricular nucleus of the hypothalamus (PVN), ventral tegmental area (VTA) and nucleus accumbens (NAc; Mountjoy, 2010; Caruso et al., 2014). Interestingly, α-MSH has also been implicated in the modulation of ethanol intake (Olney et al., 2014). The administration of a nonspecific agonist of both MC3R and MC4R (Melanotan-II) reduces voluntary alcohol intake in mice (Navarro et al., 2003) and in alcohol-preferring rats (Ploj et al., 2002). However, when this agonist was administered to knock-out mice that lack MC4R no effect was observed, revealing that MC4R (and not MC3R) is responsible for reducing alcohol intake (Navarro et al., 2011). Accordingly, the administration of a selective MC4R-agonist synthetic peptide at the NAc and VTA lowered voluntary alcohol intake in rats (Lerma-Cabrera et al., 2012). Ethanol has direct effects on the levels of α-MSH in the hypothalamus, and these effects appear to be opposite depending on whether the exposure to ethanol is chronic or acute. Evidence indicates that chronic ethanol consumption reduces α-MSH levels in the hypothalamus (Rainero et al., 1990; Navarro et al., 2008; Lerma-Cabrera et al., 2013; Sprow et al., 2016), while an increase in α-MSH was observed in acute ethanol-treated rats (Shelkar et al., 2015).

The MC system has been linked for many years with anti-inflammatory effects in the brain (Macaluso et al., 1994; Orellana et al., 2017). MC4R is expressed in microglia and astrocytes (Caruso et al., 2007; Selkirk et al., 2007; Benjamins et al., 2013), suggesting that α-MSH may produce its anti-inflammatory effects *via* MC4R in these neuroinflammation-related cells (Caruso et al., 2004). The effect of MC4R activation on the reduction of neuroinflammation has been studied in models of brain injury other than ethanol intake, e.g., LPS-induced brain inflammation (Ichiyama et al., 1999a,b; Muceniece et al., 2005), cerebral ischemia damage (Giuliani et al., 2006; Spaccapelo et al., 2011) and spinal cord injury (van de Meent et al., 1997; Lankhorst et al., 1999). There is evidence that this ability resides in its capacity to decrease glial activation of NF-κB. Specifically, α-MSH and other synthetic melanocortin receptor ligands prevent IκBα phosphorylation and, consequently, inhibit NF-κB activation (Catania, 2008). In agreement, α-MSH decreases the release of pro-inflammatory cytokines such as TNF-α (Rajora et al., 1997; Wong et al., 1997; Delgado et al., 1998; Giuliani et al., 2006; Forslin Aronsson et al., 2007; Spaccapelo et al., 2011), enzymes as iNOS and COX-2 (Caruso et al., 2004) and inflammation mediators, like NO (Galimberti et al., 1999; Muceniece et al., 2006) in the brain. In addition to the inhibition of NF-κB activity, α-MSH also stimulates the production of the anti-inflammatory cytokines IL-10 from microglia and TGF-β from astrocytes, mediated by an increased expression of peroxisome proliferator-activated receptor-gamma (PPARγ; Carniglia et al., 2013). In recent work, we demonstrated that the activation of MC4R by a synthetic agonist peptide inhibits ethanol-induced neuroinflammation in rats (Flores-Bastías et al., 2019). However, the mechanism underlying this effect has not yet been elucidated.

Although we hypothesized that the activation of MC4R in the brain inhibits NF-κB activity and neuroinflammation induced by ethanol (Flores-Bastías and Karahanian, 2018; Flores-Bastías et al., 2019), MC4R elicits additional mechanisms that would help explain the effects of α-MSH or its synthetic analogs in reducing alcohol intake. After the pioneering work of Xu et al. (2003) who reported that the activation of MC4R induces brain-derived neurotrophic factor (BDNF) expression in the hypothalamus, it was determined that BDNF is a downstream effector of MC4R signaling in food intake control (Nicholson et al., 2007; Bariohay et al., 2009). Such signaling occurs through the cAMP-PKA-CREB pathway (Caruso et al., 2012); MC4R is a G protein-coupled receptor that activates adenylate cyclase leading to an increase in cyclic AMP (cAMP) levels, which activates protein kinase A (PKA). PKA then phosphorylates and activates the cAMP-responsive element-binding protein (CREB), which is a transcription factor. CREB mediates in several physiological processes in the central nervous system by the induction of different genes, including BDNF (Tao et al., 1998). Accordingly, CREB is activated by α-MSH-treatment in the hypothalamus (Sarkar et al., 2002; Sutton et al., 2005; Caruso et al., 2010).

BDNF is a neurotrophic factor that plays several roles in neurons growth and function. BDNF, through its receptor tropomyosin-related kinase B (TrkB), regulates CREB phosphorylation *via* the extracellular-signal-regulated kinases (Erk1/2) pathway (Bibel and Barde, 2000; Schinder and Poo, 2000), playing a key role in synaptic plasticity. BDNF signaling has also been implicated in alcohol drinking behavior, dependence and withdrawal (Davis, 2008), and its levels are altered in alcoholics (Heberlein et al., 2010). A BDNF gene polymorphism has been linked to a greater predisposition to develop AUD in humans (Uhl et al., 2001), and a deficiency of the Bdnf gene causes increased alcohol intake in mice (Hensler et al., 2003). Accordingly, siRNA-mediated downregulation of endogenous BDNF in the striatum increased alcohol self-administration in rats, and subsequent infusion of exogenous BDNF reverted this response (Jeanblanc et al., 2009). It was suggested that BDNF would serve as a homeostatic factor in the hippocampus and striatum that somehow regulates ethanol intake (McGough et al., 2004). Changes in BDNF expression in the brain following chronic ethanol exposure play a role in the regulation of protracted alcohol consumption and withdrawal-induced anxiety (Pandey et al., 2006). Similar to that described above for α-MSH, exposure to ethanol has opposite effects on BDNF levels depending on whether the exposure is chronic or acute: after chronic alcohol consumption, BDNF gene expression in corticostriatal areas is downregulated (Logrip et al., 2009; Melendez et al., 2012). However, in the case of acute alcohol exposure, BDNF levels are increased in striatal and hippocampal brain regions (McGough et al., 2004; Jeanblanc et al., 2009; Logrip et al., 2009). Thus, it is conceivable that prolonged exposure to ethanol reduces α-MSH/MC4R signaling which would decrease BDNF expression, eventually leading to the establishment of a protracted alcohol consumption behavior.

What would be the mechanisms by which BDNF could regulate alcohol consumption? As mentioned above, neuroinflammation induced by ethanol is a process associated with the perpetuation of chronic alcohol intake, and the inhibition of such neuroinflammation lowers voluntary alcohol intake in animal models (Bell et al., 2015; Israel et al., 2017; Ezquer et al., 2018). Interestingly, BDNF triggers important anti-inflammatory and anti-apoptotic mechanisms in glial cells and neurons, respectively, and this could be one of the actions by which BDNF reduces chronic alcohol intake. It was reported that BDNF treatment decreases the levels of the proinflammatory cytokines TNF-α, IL-1β and IL-6 induced by bacterial components in the brain and increases the expression of the anti-inflammatory cytokine IL-10 (Xu et al., 2017). Similarly, BDNF suppressed TNF-α expression while increased IL-10 expression in the brain after ischemic injury (Jiang et al., 2011). Chronic alcohol consumption, in addition to producing neuroinflammation as we have already described, also leads to other important changes in the brain such as a reduction of neurogenesis in the hippocampus (Herrera et al., 2003; Crews et al., 2006; Zou and Crews, 2010). The hippocampus has been extensively examined because of its role in memory consolidation, along with the deterioration of cognitive function seen in alcoholics; interestingly, recovery from alcoholism is associated with increased neurogenesis in this brain region (Crews and Nixon, 2009). Because BDNF plays a fundamental role in neuroinflammation and neurogenesis, it is possible to speculate that activation of MC4R leads to the upregulation of BDNF in glia, which would decrease the neuroinflammation response (Figure 1). Also, BDNF secreted by glial cells would activate TrkB signaling in neurons, through the MAPK/ERK and PLCγ/IP3 pathways that upregulate CREB, leading to increased neurogenesis and enhanced long-term potentiation (LTP), which were reduced by chronic alcohol consumption (Figure 1). The latter would contribute significantly to improve cognitive disorders associated with alcohol consumption. Finally, BDNF signaling in neurons also activates the PI3/Akt pathway, which inhibits the pro-apoptotic protein Bad, thus decreasing ethanol-induced apoptosis and increasing neuronal survival (Figure 1). Additionally, the activation of MC4R in neurons would lead to an increased expression of BDNF through the PKA/CREB pathway in these cells. In this way, α-MSH/MC4R signaling increases BDNF secretion by neurons themselves, which would bind to their own TrkB receptors creating a self-activation loop that will potentiate the described neuroprotection mechanisms.

**Figure 1:**
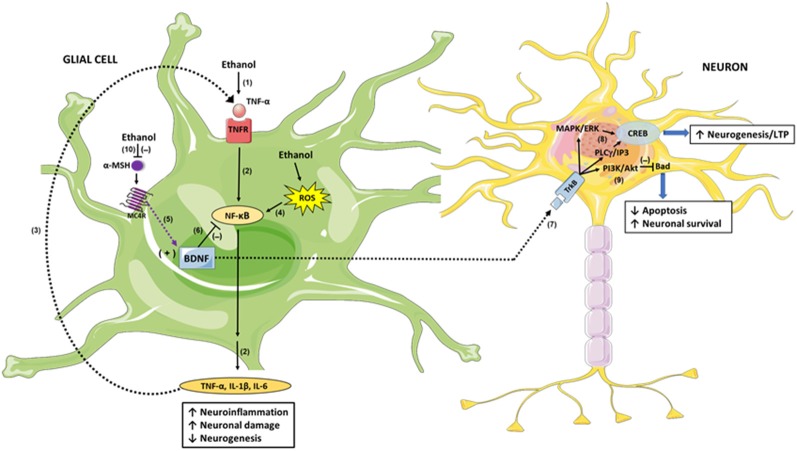
Brain-derived neurotrophic factor (BDNF) mediation in the anti-inflammatory and neuroprotective response of α-melanocyte-stimulating hormone/melanocortin 4 receptor α-MSH/MC4R against damage produced by excessive alcohol consumption. Ethanol intake increases hepatic tumor necrosis factor-α (TNF-α) release into the blood (1); TNF-α is then transported across the blood-brain-barrier to glial cells and signals through Tumor necrosis factor receptor/Nuclear factor-κB (TNFR/NF-κB), inducing the expression of inflammation mediators such as IL-1β, IL-6 and TNF-α itself (2); these proinflammatory cytokines produce neuronal damage and reduce neurogenesis. The glial-secreted TNF-α binds to TNFR, creating an activation loop that potentiates the initial neuroinflammation response (3). Additionally, ethanol-generated reactive oxygen species (ROS) activates NF-κB directly, exacerbating the inflammatory damage (4). α-MSH/MC4R signaling induces BDNF expression (5), and BDNF inhibits NF-κB activity (6) thus inhibiting the neuroinflammatory response. In addition, BDNF secreted by glial cells acts in neurons through its TrkB receptor (7), signaling through MAPK/ERK and PLCγ/IP3 pathways (8) which in turn activate CREB, leading to increased neurogenesis and enhanced long-term potentiation (LTP). Additionally, BDNF/TrkB signaling activates PI3K/Akt pathway which inhibits the pro-apoptotic protein Bad, increasing neuronal survival (9). Ethanol decreases the activity of the central melanocortins system, reducing α-MSH levels and therefore MC4R activity (10); thereby, BDNF expression is downregulated averting its anti-inflammatory and neuroprotective activities.

In summary, based on the background presented, we hypothesize that ethanol exposure during adolescence decreases the expression of α-MSH and hence MC4R signaling in the hippocampus, leading to a lower BDNF activity that causes dramatical changes in the brain (e.g., neuroinflammation, neuronal death and decreased neurogenesis) that predispose to maintain alcohol abuse until adulthood. The activation of MC4R either by α-MSH or by synthetic agonist peptides is able to induce the expression of BDNF, which would trigger several processes that lead to lower both neuroinflammation and alcohol consumption in adulthood.

## Author Contributions

OF-B and EK wrote the article. AA-C designed the figure.

## Conflict of Interest

The authors declare that the research was conducted in the absence of any commercial or financial relationships that could be construed as a potential conflict of interest.
